# Factors associated with incomplete surgical margins in basal cell carcinoma of the head and neck

**DOI:** 10.1016/j.bjorl.2020.02.007

**Published:** 2020-04-08

**Authors:** Fábio Muradás Girardi, Vivian Petersen Wagner, Manoela Domingues Martins, Aliende Lengler Abentroth, Luiz Alberto Hauth

**Affiliations:** a Hospital Ana Nery, Centro Integrado de Oncologia, Santa Cruz do Sul, RS, Brazil; b Universidade de Campinas, Faculdade de Odontologia de Piracicaba, Departamento de Diagnóstico Oral, Piracicaba, SP, Brazil; c Universidade Federal do Rio Grande do Sul (UFRGS), Faculdade de Odontologia, Departamento de Patologia Oral, Porto Alegre, RS, Brazil; d Universidade Federal do Rio Grande do Sul (UFRGS), Hospital de Clínicas de Porto Alegre (HCPA), Departamento de Medicina Oral, Porto Alegre, RS, Brazil

**Keywords:** Carcinoma, basal cell, Margins of excision, Head and neck neoplasms, Cohort studies

## Abstract

**Introduction:**

Cutaneous basal cell carcinoma recurrence is associated with inadequate surgical margins. The frequency of and the factors associated with compromised or inadequate surgical margins in head and neck basal cell carcinoma varies.

**Objective:**

The purpose of this study was to evaluate the clinical and pathological factors associated with inadequate surgical margins in head and neck basal cell carcinoma.

**Methods:**

We developed a cross-sectional study comprising all patients who had undergone resection of head and neck basal cell carcinoma from January 2017 to December 2019. Data on age, sex, head and neck topography, histopathological findings, and staging were retrieved and compared. Each tumor was considered an individual case. Compromised and close margins were termed “inadequate” or “incomplete”. Variables that were significantly associated with the presence of incomplete margins were further assessed by logistic regression.

**Results:**

In total, 605 tumors from 389 patients were included. Overall, sixteen cases (2.6%) were classified as compromised, 52 (8.5%) as close, and 537 (88.7%) as free margins. Presence of scleroderma (*p* = 0.005), higher Clark level (*p* < 0.001), aggressive variants (*p* < 0.001), invasion beyond the adipose tissue (*p* < 0.001), higher T stage (*p* < 0.001), perineural invasion (*p* = 0.002), primary site (*p* = 0.04), multifocality (*p* = 0.01), and tumor diameter (*p* = 0.02) showed association with inadequate margins. After Logist regression, multifocality, Clark level and depth of invasion were found to be independent risk factors for inadequate margins.

**Conclusion:**

Gross clinical examination may be sufficient for determining low prevalence of inadequate surgical margins when treating head and neck basal cell carcinoma in highly experienced oncologic centers. Multifocality, Clark level and depth of invasion were found to be independent risk factors for incomplete margins.

## Introduction

Basal cell carcinoma (BCC) is a disease that mainly affects the head and neck of fair-skinned individuals with a history of sun exposure throughout life. Most cases occur in individuals after the sixth decade of life, with a slight tendency in males.^1^ BCC are usually slow-growing tumors, which are rarely associated with death. The metastatic potential of these lesions is restricted to a few cases.^2^ When death related to the disease occurs, most are due to unresectability, inoperability, perioperative mortality in high-risk surgeries, or rare cases of regional or distant metastasis.^2^, ^3^ In the head and neck region, these features might be present in lesions of the upper middle third of the face, with infiltrative disease at the base of the skull.^3^

Recurrence is also associated with major rates of distant disease and larger resections, being directly related to inadequate surgical margins.^2^, ^4^, ^5^ The definition of appropriate operative margins for BCC usually varies according to the size of the lesion and its location. Most guidelines recommend macroscopic clinical margins of 2–4 mm.^1^, ^6^ In histopathological analysis, cases of complete resection are those with microscopic neoplasm-free margins of at least 1 mm. Although relapses may be observed in cases with larger microscopic surgical margins, the risk of the event is four times lower when compared to cases of incomplete resection.^7^

In the medical literature, the frequency of compromised or inadequate surgical margins in BCC varies. In the head and neck region, the prevalence of positive margins varies from 9% to 37.2%.^8^, ^9^, ^10^, ^11^, ^12^ Head and neck tumors, especially in some topographic subregions and among less experienced professionals, appear to be associated more with compromised or inadequate margins compared to BCC in other sites.^11^, ^12^, ^13^, ^14^ Herein, our main objective was to evaluate the clinical and pathological factors associated with inadequate surgical margins in head and neck BCC through an institutional series in a tertiary oncological center.

## Methods

The Research Ethics Committee approved the present study (CAAE: 93792318.4.0000.5304). We developed a cross-sectional study comprising all patients who had undergone resection of malignant neoplasms of the skin of the head and neck from January 2017 to December 2019. Data on age, sex, topography, histopathological findings, and staging were retrieved and included in a specific database. The classification of BCC cases was made according to Rosai (2004) in: nodular, superficial, infiltrating, micronodular, fibroepithelial, basosquamous, keratotic, pigmented, infundibulocystic, adenoid, cystic, sclerosing, and clear cell.^15^ Cases that underwent only margin enlargement (33 cases), or with a diverse histology of BCC (266 cases with squamous cell carcinoma, one case with Merkel cell carcinoma, 3 cutaneous sarcomas, and 15 cases with melanoma) were excluded from the analysis.

The same head and neck surgeon (FMG) treated all patients, and all surgeries were performed using conventional techniques, without intraoperative pathological analysis. Prior to excision, all tumors were visually inspected through the naked eye to ensure macroscopically free margins of at least 0.2–0.4 cm, according to the tumor diameter, as stated by the current UK Guidelines.^6^

The tumors were staged pathologically according to the eighth edition of the American Joint Committee on Cancer (AJCC) pathological Tumor-Node-Metastasis (pTNM) staging system.^16^ The tumor diameter and depth of invasion (DOI) were expressed in cm. Margins were considered compromised, close (<0.1 cm), or free (≥0.1 cm) according to the histopathological analysis findings. Compromised and close margins were considered “inadequate” or “incomplete”. Cases were considered aggressive if classified microscopically as micronodular, infiltrating, fibroepithelial, or basosquamous. All the other microscopic subtypes were considered non-aggressive.^17^ The head and neck topographic regions were divided in: forehead; brown; periorbital; temple; zygomatic; infraorbital; nasal; ear; upper lip; mandibular; lower lip; chin; cervical; scalp; and retroauricular. When comparing primary site, tumors in the nose, lip, ear, and periorbital zone were termed “noble parts” and compared together to the other parts of the face and neck. Each tumor was analyzed as an individual case for comparison between groups. The only exception was margin status evaluation according to the number of tumors resected in the same patient, where the patient was considered the unit of comparison.

The data were summarized using descriptive analysis. Continuous variables with normal distribution are expressed as the mean and standard deviation. Variables with non-normal distribution are expressed as the median, minimum, and maximum. Categorical variables are expressed as the absolute and relative frequency. The association of surgical margin status and continuous variables was assessed using Kruskal–Wallis or Mann–Whitney *U* test. The Chi-Square test and Fischer exact test were used for categorical variables. Variables that were significantly associated with the presence of inadequate margins were assessed using logistic regression to calculate the Odds Ratio (OR) and 95% Confidence Interval in a univariable Model. Next, the variables were incorporated into a multivariable model and the stepwise backward method was used to achieve a final model in which variables with *p* < 0.10 were maintained. The statistical analysis was performed using SPSS software version 20.0 (SPSS Inc., Chicago, IL). All tests considered a level of significance of 5%.

## Results

Of 923 resected skin tumors, we included 605 excised from 389 patients. The mean patient age was 69 ± 11.45 years, with a slight predominance of male patients (52.8%). The most common topography was nasal (122 cases, 20.2%), followed by infraorbital zone (84 cases, 13.9%). The remaining topographies and rates are found in Table 1. The most common histological type was nodular (382 cases, 63.1%), followed by basosquamous (57 cases, 9.4%). 136 cases (22.4%) had more than one tumor variant. Lateral margins were compromised, close, and free in 12 (1.9%), 29 (4.7%), and 564 cases (93.2%), respectively. The deep margins were millimetric specified in only 207 cases (34.2%) cases; 6 cases (0.9%) were compromised, and 29 cases (4.7%) were close. Overall, based in both the lateral and deep margins, 16 cases (2.6%) were classified as compromised, 52 (8.5%) as close, and 537 (88.7%) as free margins. These data were used for statistical analysis.

**Table 1:** Topographic distribution of BCC cases according to margin status.

Site		Margins		
		Compromised	Close	Free
Forehead	*n*	3	2	43
	%	18.8	3.8	8.0%
Brown	*n*	0	0	9
	%	0.0	0.0	1.7%
Periorbital	*n*	2	5	61
	%	12.5	9.6	11.4%
Temple	*n*	1	5	43
	%	6.2	9.6	8.0%
Ear	*n*	1	4	41
	%	6.2	7.7	7.6%
Zygomatic	*n*	2	1	20
	%	12.5	1.9	3.7%
Infraorbital	*n*	1	9	74
	%	6.2	17.3	13.8%
Nasal	*n*	5	14	103
	%	31.2	26.9	19.2%
Upper Lip	*n*	0	3	19
	%	0.0	5.8	3.5%
Lower Lip	*n*	1	2	4
	%	6.2	3.8	0.7%
Mandibular	*n*	0	3	35
	%	0.0	5.8	6.5%
Chin	*n*	0	0	6
	%	0.0	0.0	1.1%
Cervical	*n*	0	1	58
	%	0.0	1.9	10.8%
Scalp	*n*	0	2	15
	%	0.0	3.8	2.8%
Retroarticular	*n*	0	1	6
	%	0.0	1.9	1.1%
Total	*n*	16	52	537
	%	100.0	100.0	100.0%

We found a significant association between compromised margins and more aggressive variants (*p* < 0.001), higher Clark level (*p* < 0.001), higher prevalence of invasion beyond the adipose tissue (*p* < 0.001), higher T stage (*p* < 0.001), presence of scleroderma (*p* < 0.001), perineural invasion (*p* < 0.001) and DOI (*p* < 0.001) (Table 2). No association was observed for multiple tumors operated within the 3 year study period (*p* = 0.14), sex (*p* = 0.14), age (*p* = 0.23), type of reconstruction (*p* = 0.38), angiolymphatic invasion (*p* = 0.40), ulceration (*p* = 0.53), and primary site (*p* = 0.17). There was a trend indicating association between compromised margins and multifocality (*p* = 0.05), and tumor diameter (*p* = 0.06), although with no statistical significance. Analyzing adequate versus inadequate margins, not only presence of scleroderma (*p* = 0.005), higher Clark level (*p* < 0.001), aggressive variants (*p* < 0.001), invasion beyond the adipose tissue (*p* < 0.001), higher T stage (*p* < 0.001), and perineural invasion (*p* = 0.002) showed statistical association with inadequate margins, but also primary site (*p* = 0.04), multifocality (*p* = 0.01), and tumor diameter (*p* = 0.02) (Table 3). Stepwise regression analysis was used to assess the independent factors associated to inadequate margins. Table 3 summarizes the odds ratios and confidence intervals of the factors which showed statistical significance in multivariate analysis. The set included all variables analyzed. Multifocality, Clark level and DOI were found to be independent risk factors for inadequate margins.

**Table 2:** Association of BCC clinic–pathologic features and margin status.

	Compromised		Close		Free		*p*-value^a^
	*n* = 16	%	*n* = 52	%	*n* = 537	%	
*Gender*							0.14
M	6	37.5	33	53.7	281	52.3	
F	10	62.5	19	63.5	256	47.7	

*Site*							0.17
Noble sites^b^	9	56.2	28	53.8	228	42.5	
Others	7	43.8	24	46.2	309	57.5	

*Reconstruction*							0.38
Flap/Z plasty	4	25.0	15	28.8	137	25.5	
Primary suture	8	50.0	28	53.8	336	62.6	
Graft	4	25.0	9	17.3	64	11.9	

*Scleroderma*	7	43.8	6	11.5	43	8.0	<0.001
*Multifocality*	6	37.5	16	30.8	107	19.9	0.05

*Clark*							<0.001
I–III	4	25.0	19	37.3	317	60.8	
IV–V	12	75.0	32	62.7	204	39.2	

*Ulcer*							0.53
Present	9	60.0	14	35.0	182	42.3	
Erosion	2	13.3	11	27.5	92	21.4	
Absent	4	26.7	15	37.5	156	36.3	

*Variant risk*							<0.001
High	9	56.2	20	38.5	95	17.7	
Low	7	43.8	32	61.5	442	82.3	

*Invasion beyond adipose tissue*	4	25.0	9	17.3	24	4.5	<0.001
*PN invasion*	2	12.5	6	11.5	15	2.8	<0.001
*AL invasion*	1	6.2	2	3.8	11	2.0	0.40

*T stage*							<0.001
T1–2	10	62.5	36	72.0	490	92.8	
T3–4	6	37.5	14	28.0	38	7.2	

*Age, mean (SD)*	72.9 (10.1)		70.1 (10.9)		68.8 (11.5)		0.23
*Diameter, median (range)*	0.9 (0.4–5.5)		0.95 (0.3–4.2)		0.8 (0.05–8.5)		0.06
*DOI, median (range)*	0.21 (0.1–4.0)		0.2 (0.05–1.7)		0.15 (0.0–1.8)		0.004

Data expressed in absolute and relative values.

M, male; F, female; PN, perineural; AL, angiolymphatic; age expressed in years. DOI, depth of invasion; diameter and DOI expressed in cm; SD, standard deviation; range, minimum and maximum values; *p*, level of significance.

a Chi-Square test for categorical variables and Kruskal–Wallis test for continous variables.

b Noble sites = nose, lips, eyelid and ear.

**Table 3:** Predictors of BCC inadequate margins.

	Inadequate		Adequate		*p*-value^a^	Univariable logistic regression		Multivariable logistic regression	
	*n* = 16	%	*n* = 537	%		OR (95% IC)	*p*-value	OR (95% IC)	*p*-value
*Gender*									
M	39	57.4	281	52.3	0.25				
F	29	42.6	256	47.7					

*Site*									
Noble sites^b^	37	54.4	228	42.5	0.04	1.61 (0.97–2.68)	0.06		
Others	31	45.6	309	57.5					

*Reconstruction*									
Flap/Z plasty	19	27.9	137	25.5	0.17				
Primary suture	36	52.9	336	62.6					
Graft	13	19.1	64	11.9					

*Scleroderma*	13	19.1	43	8.0	0.005	2.71 (1.36–5.36)	0.004		
*Multifocality*	22	32.4	107	19.9	0.01	1.92 (1.10–3.33)	0.02	5.33 (2.54–11.20)	<0.001

*Clark*									
I–III	23	34.3	317	60.8	<0.001	2.97 (1.74–5.07)	<0.001	2.60 (1.27–5.31)	0.009
IV–V	44	65.7	204	39.2					

*Ulcer*									
Present	23	41.8	182	42.3	0.92				
Erosion	13	23.6	92	21.4					
Absent	19	34.5	156	36.3					

*Variant risk*									
High	29	42.6	95	17.7	<0.001	3.46 (2.03–5.87)	<0.001		
Low	39	57.4	442	82.3					

*Invasion beyond adipose tissue*	13	19.1	24	4.5	<0.001	5.05 (2.43–10.48)	<0.001		
*PN invasion*	8	11.8	15	2.8	0.002	4.64 (1.88–11.39)	0.001		
*AL invasion*	3	4.4	11	2.0	0.20				

*T stage*									
T1–2	46	69.7	490	92.8	<0.001	5.60 (3.01–10.42)	<0.001		
T3–4	20	30.3	38	7.2					

*Age, mean (SD)*	70.8 (10.7)		68.8 (11.5)		0.23				
*Diameter, median (range)*	0.9 (0.3–5.5)		0.8 (0.05–8.5)		0.02	1.48 (1.31–1.94)	0.004		
*DOI, median (range)*	0.2 (0.05–4.0)		0.15 (0.0–1.8)		0.004	5.87 (1.8–18.42)	0.002	5.44 (1.54–19.12)	0.008

Data expressed in absolute and relative values.

M, male; F, female; PN, perineural; AL, angiolymphatic; age expressed in years. DOI, depth of invasion; diameter and DOI expressed in cm; SD, standard deviation; range, minimum and maximum values; OR, odds ratio; CI, confidence interval; *p*, level of significance.

a Fisher exact test for categorical variables and Mann–Whitney test for continous variables.

b Noble sites = nose, lips, eyelid and ear.

## Discussion

In the medical literature, the frequency of inadequate surgical margins in BCC varies. Positive margins in head and neck BCC are more common compared to that of other skin sites, with 9%–37.2% prevalence of compromised margins.^8^, ^9^, ^10^, ^11^, ^12^ In our experience, 2.6% and 8.5% of cases presented compromised margins and inadequate surgical margins, respectively. It is possible that being treated by a head and neck surgeon influenced the results. Comparative studies could elucidate this finding, although different rates of incomplete surgical margin for skin cancer when comparing some surgical specialties have been shown in the literature.^14^, ^18^, ^19^ The present report is the first to include only head and neck surgeons in this scenario. This is probably because, in the majority of oncological centers, the commitment of these professionals to non-melanoma skin cancers is focused only on aggressive or recurrent cases involving deep underlying structures and major resections. We can say that the reality observed at our center is an exception, as we treat the majority of skin cancer cases in our region, even small head and neck lesions. Transposing to the reality of oral cancers, Hanasono et al. showed that advanced cases reconstructed with microsurgical flaps have a lower prevalence of compromised margins when compared to those of smaller diameter treated with other forms of reconstruction. The authors suggested that larger resections could be performed knowing that more extensive defects could be reliably reconstructed. In other words, the availability and the institutional ability to perform the reconstruction might interfere with the surgeon's “freedom” at the time of tumor resection.^20^

Complete tumor resection is one of the major prognostic factors in head and neck oncology^21^ and this is not different among cutaneous neoplasms.^3^ Recurrences are associated with worse outcomes, being directly related to inadequate surgical margins.^2^, ^5^, ^12^ Codazzi et al. found that about 25% of skin cancer cases with incomplete resection recurred, while only 6% of completely resected cases presented with recurrence.^7^ Godoy et al. found an association between fibrosing-type BCC and increased risk of inadequate margins.^13^ Cho et al. also found an association between inadequate margins, more aggressive BCC variants, and perineural invasion.^22^ Studying BCCs from all parts of the body, Codazzi et al. observed a higher prevalence of incomplete margins in cases with head and neck disease, recurrent tumors with greater DOI, more aggressive variants, and advanced age.^7^ Farhi et al. identified a higher frequency of incomplete margins in nasal BCCs, those from the inner corner of the eye, and in more infiltrative and in multifocal tumors.^23^ Here, we observed that inadequate surgical margins were more frequent if there was a higher prevalence of invasion beyond adipose tissue, perineural invasion, higher T stage, diameter and DOI, factors considered for the increase of the pathological stage according to the eighth edition of the AJCC staging system. It is likely that the same factors are also involved with a greater chance of recurrence, corroborating with the updates of the current staging system. Multifocality, presence of tumor in noble zones, and presence of scleroderma also confer to the tumor an increased risk of inadequate surgical margins. The indefinition of the tumor borders and the limits of resection without significantly compromising function and esthetics probably influenced those results.

There are different means of reducing the risk of involvement of the surgical margins in cutaneous tumors. The most accepted is Mohs micrographic surgery, currently considered the gold standard for cutaneous oncologic surgery.^24^ This surgery is not routine at our tertiary center. The method is not covered by the public or even by the private health care providers in our country, which are the financial resource for the majority of our patients’ treatments. Ours is the reference center for a region with a high incidence of skin cancer, with a Caucasian population actively linked to agrarian activities. Keeping the same rates of relapse observed by Codazzi et al.^7^ and our rates of compromised and incomplete margins, our estimate is 4–17 reoperations per year directly related to margin status. We have not observed difficulties in the surgical rescue of patients with recurrence, and we advocate that, for centers with a high volume of surgeries and a low rate of inadequate surgical margins, it is possible for surgical practice to remain conservative.

The main limitation of our study is the low volume of cases with compromised margins, compensated by dividing the sample in adequate and inadequate margins. Another possible limitation is the loss of millimetric discrimination at the deep margins in a significant portion of cases, although they were described as free. It is important to stress that in some particular areas, close deep margins but with preservation of the underlying tissue may be appropriate. A classical example is those cases of skin cancer of the auricular concha, scapha or antihelix, with no signs of cartilage infiltration, where resection of perichondrium may be sufficient and adequate to preserve esthetics and functionality. In other areas where the subcutaneous adipose tissue is generous, it is a common practice to excise a thick sample of deep tissue even in initial skin tumors. Studies of follow-up or analysis of the pattern of recurrent cases could help resolve this issue.

## Conclusion

Gross clinical examination may be sufficient for resulting in a low prevalence of inadequate surgical margins when treating head and neck BCC at highly experienced oncologic centers. Multifocality, Clark level and DOI were found to be independent risk factors for inadequate margins.

## Ethical approval

All procedures performed in studies involving human participants were in accordance with the ethical standards of the institutional and/or national research committee and with the 1964 Helsinki declaration and its later amendments or comparable ethical standards.

## Informed consent

As this study was retrospective and with no intervention, no informed consent was applied.

## Conflicts of interest

The authors declare no conflicts of interest.
